# Preoperative radiotherapy does not improve and may even be detrimental to the long-term prognosis of patients diagnosed with stage III colon adenocarcinoma: a propensity score-matched SEER database analysis

**DOI:** 10.3389/fonc.2023.1324485

**Published:** 2023-11-22

**Authors:** Jinyi Xu, Xiaoqiang Niu

**Affiliations:** ^1^ Department of Gastrointestinal Surgery, The Second Affiliated Hospital of Nanchang University, Nanchang, China; ^2^ Jiangxi Medical College, Nanchang University, Nanchang, China

**Keywords:** preoperative radiotherapy, colon adenocarcinoma, SEER, overall survival, propensity score matching

## Abstract

**Background:**

Currently, for patients with colon adenocarcinoma who are diagnosed with local lymph node metastasis, it is typically recommended to undergo neoadjuvant treatment before undergoing curative surgical intervention. Nowadays, the focus of preoperative adjuvant therapy for colon adenocarcinoma patients mainly revolves around chemotherapy, and the impact of preoperative radiotherapy on long-term prognosis remains uncertain.

**Methods:**

We extracted data from the Surveillance, Epidemiology, and End Results database for patients with stage III colon adenocarcinoma between 2004 and 2019. Using propensity score matching (PSM), the patients were divided into a preoperative radiotherapy group and a non-preoperative radiotherapy group, and the differences in Kaplan-Meier (KM) survival curves between the two groups were compared. Cox regression analysis was employed to identify clinical factors that influence survival in stage III colon adenocarcinoma, and the prognostic differences between the two groups were compared within specific subgroups of these clinical factors.

**Results:**

After PSM, a total of 242 patients were included in the study, divided into the preoperative radiotherapy group and the non-preoperative radiotherapy group. There were no statistically significant differences in important clinical characteristics between the two groups. KM analysis revealed no statistically significant difference in overall survival (OS) between the two groups. Furthermore, age, chemotherapy, T staging, N staging, race, tumor grade, gender, tumor location, and tumor diameter were identified as important factors influencing the prognosis of patients. Within each level of the aforementioned subgroups, there were no differences in OS between the two groups. In fact, in specific subgroups, the non-preoperative radiotherapy group exhibited better OS than the preoperative radiotherapy group.

**Conclusion:**

Preoperative radiotherapy does not improve the long-term prognosis of patients with stage III colon adenocarcinoma. In certain patient populations with specific clinical characteristics, preoperative radiotherapy may even lead to a decrease in OS.

## 1 Introduction

Colorectal cancer is one of the most prevalent malignant tumors worldwide. Recent global cancer statistics have shown that the incidence of colorectal cancer has risen to the third highest, with the mortality rate ranking second, and the number of newly diagnosed cases ranking fifth (1). Currently, adjuvant chemotherapy following curative surgery remains the preferred curative treatment for colorectal cancer (2). However, due to population aging and urban industrialization, the incidence and mortality rates of colorectal cancer have significantly increased (3). Moreover, an increasing number of colorectal cancer patients are being diagnosed with regional lymph node metastasis (stage III according to AJCC staging), which further complicates effective treatment.

In recent years, more researchers believe that patients with lymph node metastasis at the time of preoperative diagnosis should consider receiving neoadjuvant therapy in order to reduce tumor staging, improve R0 resection rate, decrease local recurrence rate, and achieve clinical complete response (cCR) or even pathological complete response (pCR) for some patients (4–6).

However, current research on preoperative neoadjuvant therapy for colorectal cancer mainly focuses on chemotherapy, while the safety of adjuvant radiotherapy and its impact on long-term prognosis still remain controversial (4, 7, 8). In this study, we selected stage III colon adenocarcinoma (CA) patients diagnosed between 2004 and 2019 from the Surveillance, Epidemiology, and End Results (SEER) database to determine the long-term survival benefits of preoperative radiotherapy. We also conducted comparative analyses within different subgroups to explore characteristics of populations that may benefit from preoperative radiotherapy.

## 2 Materials and methods

### 2.1 Data source

The dataset of CA patients in this study is derived from the SEER database. Patients were selected based on the World Health Organization’s International Classification of Diseases, Third Edition (ICD-3) codes (8140-8389) for pathologically diagnosed primary colon adenocarcinoma from 2004 to 2019. Data including age, sex, race, tumor size, tumor differentiation, tumor location, tumor staging, surgery, preoperative radiotherapy (RBS), chemotherapy, and survival period (survival time and status) were extracted from the SEER database.

### 2.2 Patient selection criteria

This study included patients who met the following criteria: (1) underwent curative surgery, (2) were classified as stage III according to AJCC staging, and (3) were pathologically diagnosed with CA. Patients were excluded from this study if they met any of the following criteria: (1) diagnosed through autopsy or based on death certificates, (2) had unknown clinical data, or (3) had a survival time of less than one month. Based on whether patients received radiotherapy before surgery (RBS), they were divided into two groups: the surgery group (None-RBS) and the radiotherapy before surgery group (RBS).

### 2.3 Outcome variable and covariates

The primary outcome variable in our study is overall survival (OS) of patients. OS is defined as the time from the date of diagnosis to the date of patient’s death or last follow-up. Additionally, we selected several clinical covariates that are closely associated with OS in colorectal cancer patients, including age, sex, race, tumor size, tumor differentiation, tumor location, tumor staging, surgery, and chemotherapy. We stratified patients based on each covariate and constructed Cox models within each subgroup to assess the impact of preoperative radiotherapy on OS among different subgroups of patients.

### 2.4 Propensity score matching

The propensity score is defined as the likelihood of receiving RBS (within the range of 0 to 1) based on individual characteristics. It is derived from a logistic regression model that considers the independent associations of all available variables (i-x) with the RBS status (xi). In summary, a 1:1 nearest neighbor matching method was used to match baseline characteristics between the two groups, with a caliper width of 0.02 standard deviations. By comparing the survival outcomes of matched RBS and None-RBS patient groups, we aim to mitigate selection bias for specific patients receiving RBS (9, 10). The validation of PSM is achieved by comparing various observed variables between the RBS and None-RBS groups before and after PSM.

### 2.5 Statistical analyses

All statistical analyses in this study were performed using R software (version 4.3.1). All tests conducted were two-sided, and a p-value of <0.05 was considered statistically significant. The chi-square (χ2) test or Fisher’s exact test was used for comparing baseline data between the two patient groups. Overall survival (OS) analysis comparing the two groups was performed using Kaplan-Meier (K-M) method with log-rank test. Cox proportional hazards models were applied to analyze all predictor variables (i-xi) using the procedure in the MuMIn package, with Breslow approximation for handling ties. This procedure generated a set of Cox models with different combinations of variables. Within this set, we utilized an information-theoretic framework to identify the best-fitting models (11, 12). Specifically, the adjusted Akaike information criterion (AICc) was calculated, which measures the amount of information provided by a model while penalizing for overfitting. The AICc values were used to select a 95% confidence set, representing the best-approximating models that may include the true model. Hazard ratio estimates for RBS and other predictive factors within the 95% confidence set were averaged (weighted by AICc) to infer prognostic indicators for survival. Subsequently, patients were stratified within each subgroup of the identified risk factors in the best model to explore differences in OS between the two cohorts within specific stratified patient populations.

## 3 Results

### 3.1 Baseline characteristics of the study population

**Figure 1:**
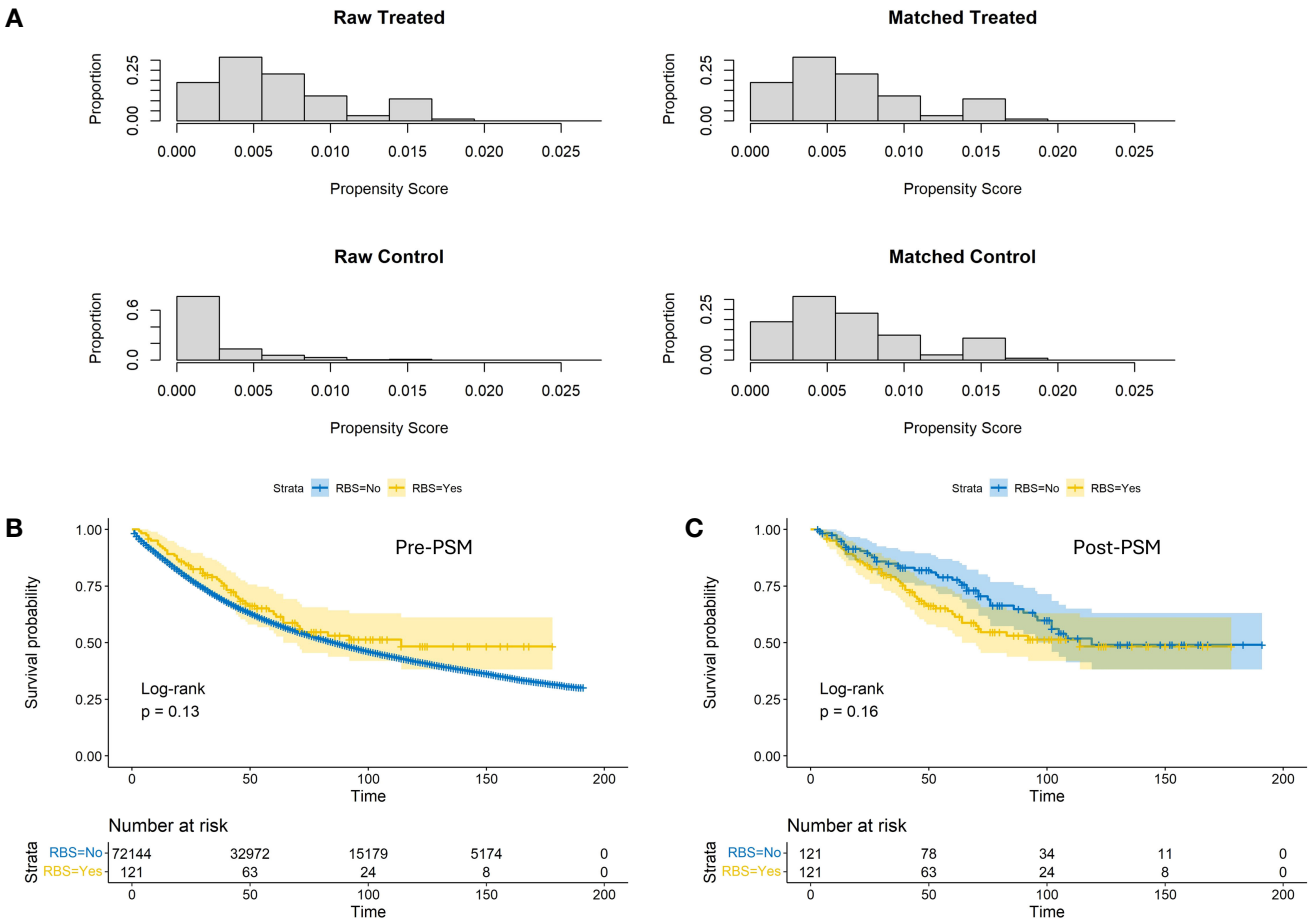
Propensity score distributions **(A)** and Kaplan-Meier estimates of OS with corresponding 95% confidence intervals before **(B)** and after **(C)** conducting propensity score matching between the RBS group and the none-RBS group.

A total of 72,365 eligible patients were included in this study, with 121 patients in the RBS group and 72,144 patients in the None-RBS group. As shown in **Table 1**, significant differences in baseline characteristics were observed between the two groups. Compared to the None-RBS group, the RBS group had a higher proportion of young patients, male patients, Grade I-II patients, left-sided colon cancer patients, and patients receiving chemotherapy (all p<0.05). However, after performing 1:1 propensity score matching (PSM) (**Figure 1A**), the baseline characteristics between the two groups became comparable (all p>0.05, **Table 1**).

**Table 1:** The baseline characteristics before and after propensity score matching reveal the statistical comparison between the RBS group (highlighted as the reference group) and the None-RBS group (chi-square test).

	Pre-PSM		RBS (N=121)	Post-PSM	
	None-RBS	Comparison		Comparison	None-RBS
	(N=72144)				(N=121)
**Age**		χ2 = 22.15		χ2 = 0.09	
<60	21105 (29.3%)	p<0.001	55 (45.5%)	p=0.955	57 (47.1%)
60-70	19515 (27.1%)		37 (30.6%)		35 (28.9%)
>70	31524 (43.7%)		29 (24.0%)		29 (24.0%)
**Sex**		χ2 = 8.88		χ2 = 0.00	
Female	36905 (51.2%)	p=0.003	45 (37.2%)	p=1.000	44 (36.4%)
Male	35239 (48.8%)		76 (62.8%)		77 (63.6%)
**Race**		χ2 = 3.10		χ2 = 0.00	
White	56221 (77.9%)	p=0.213	90 (74.4%)	p=1.000	90 (74.4%)
Black	8648 (12.0%)		13 (10.7%)		13 (10.7%)
Other	7275 (10.1%)		18 (14.9%)		18 (14.9%)
**Grade**		χ2 = 9.94		χ2 = 0.07	
Grade I	3989 (5.5%)	p=0.019	11 (9.1%)	p=0.995	12 (9.9%)
Grade II	49408 (68.5%)		92 (76.0%)		92 (76.0%)
Grade III	16462 (22.8%)		17 (14.0%)		16 (13.2%)
Grade IV	2285 (3.2%)		1 (0.8%)		1 (0.8%)
**T stage**		χ2 = 4.10		χ2 = 0.77	
T1	2662 (3.7%)	p=0.251	4 (3.3%)	p=0.856	2 (1.7%)
T2	6507 (9.0%)		14 (11.6%)		13 (10.7%)
T3	48125 (66.7%)		71 (58.7%)		72 (59.5%)
T4	14850 (20.6%)		32 (26.4%)		34 (28.1%)
**N stage**		χ2 = 2.27		χ2 = 0.00	
N1	48731 (67.5%)	p=0.132	90 (74.4%)	p=1.000	91 (75.2%)
N2	23413 (32.5%)		31 (25.6%)		30 (24.8%)
**Tumor size**		χ2 = 5.68		χ2 = 0.00	
<30	13109 (18.2%)	p=0.058	27 (22.3%)	p=1.000	27 (22.3%)
30-50	34034 (47.2%)		44 (36.4%)		44 (36.4%)
>50	25001 (34.7%)		50 (41.3%)		50 (41.3%)
**Tumor site**		χ2 = 132.83		χ2 = 0.27	
Left-side	28636 (39.7%)	p<0.001	110(90.9%)	p=0.966	110 (90.9%)
Right-side	35664 (49.4%)		7 (5.8%)		8 (6.6%)
Transverse colon	6873 (9.5%)		3 (2.5%)		2 (1.7%)
Overlapping lesion	971 (1.3%)		1 (0.8%)		1 (0.8%)
**Chemotherapy**		χ2 = 53.96		χ2 = 0.00	
Yes	44187 (61.2%)	p<0.001	114(94.2%)	p=1.000	113 (93.4%)
No/Unknown	27957 (38.8%)		7 (5.8%)		8 (6.6%)

### 3.2 Effect of preoperative radiotherapy on OS in stage III CA patients

Prior to PSM, patients receiving RBS exhibited slightly better OS rates at various time points compared to the None-RBS group, but the difference was not statistically significant (p=0.13, **Figure 1B**). After PSM, non-RBS patients showed a trend of better early OS rates compared to RBS patients. However, as the follow-up time increased and the number of censoring events grew, the OS rates between the two groups became more consistent. Nonetheless, there was still no statistically significant difference in OS between the two groups (p=0.16, **Figure 1C**).

### 3.3 Effect of levels of various factors on OS in stage III CA patients

**Figure 2:**
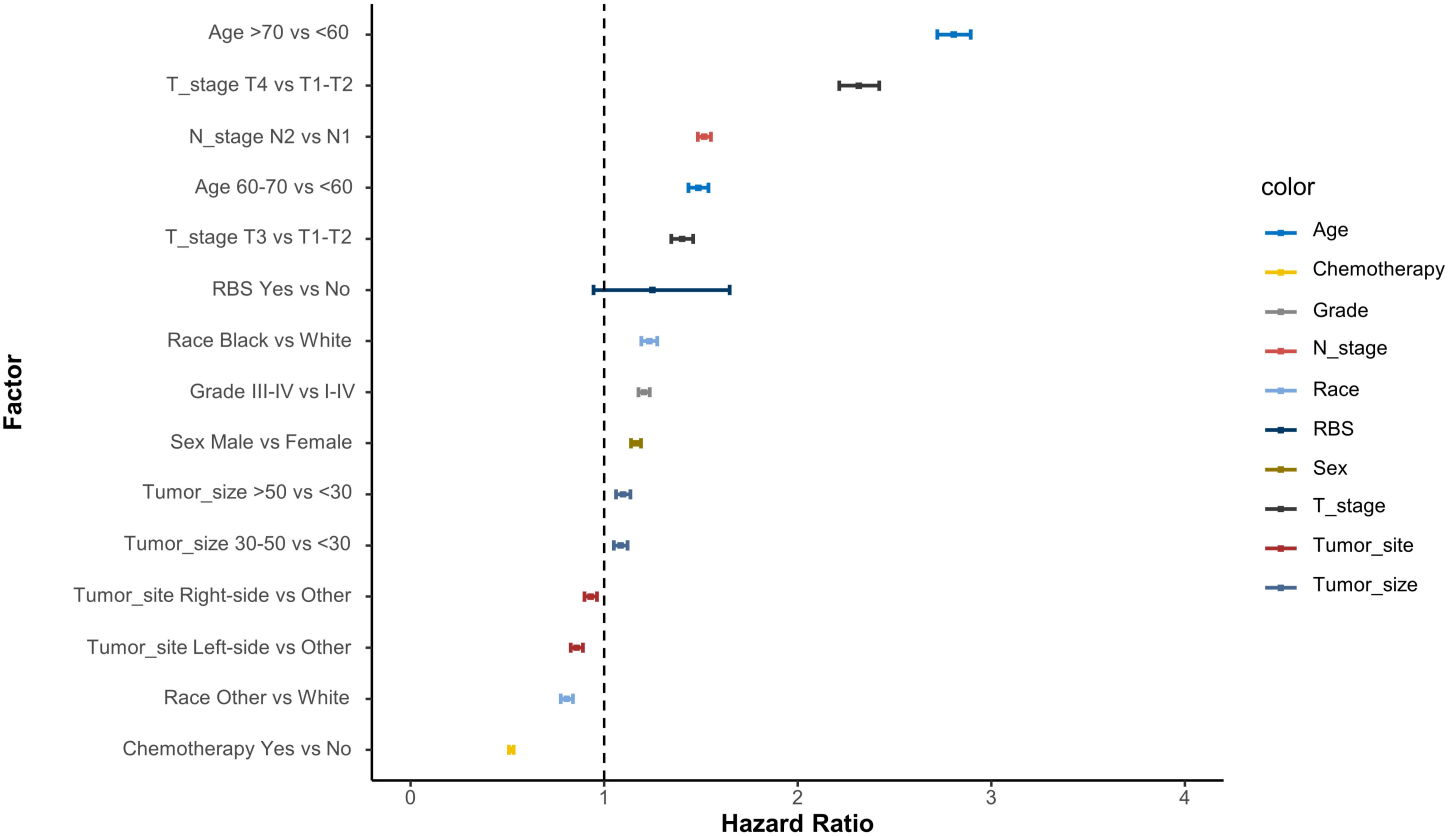
Cox proportional hazard ratios with 95% confidence intervals averaged across the model. A dashed line represents the reference hazard ratio (HR=1).

The IT-AIC method was employed to estimate the effect of RBS in a multivariable context and identify additional prognostic factors that contribute to the selection of RBS patients. According to AICc, there was no single model that clearly best explained overall survival (**Table 2**). The top-ranking models included 10 variables, with a likelihood of being the best-approximating model at 53.6%. To improve the expected predictive accuracy while maintaining low overfitting, we considered a “confidence set” consisting of two models, which together accounted for 100% likelihood of including the best model. These models indicated that the following factors were informative for predicting survival rates: (1) age, (2) chemotherapy, (3) T staging, (4) N staging, (5) race, (6) tumor grade, (7) sex, (8) tumor location, (9) tumor diameter, and (10) RBS. Based on the variables included in the models, corresponding Cox forest plots were constructed (**Figure 2**). It can be observed that advanced age, later T and N staging, and larger tumor diameter were unfavorable for the prognosis of stage III CA patients. On the other hand, receiving chemotherapy, specific tumor locations, and certain racial backgrounds were associated with improved survival time for CA patients.

**Table 2:** Set of models created with cox stepwise regression, ranked by corrected AIC.

Age	Chm	T	N	Race	Grd	Sex	Site	Size	RBS	K	LL	AICc	ΔAIC	AICcW
										10	-345485.5	691001.1	0.00	0.536
										9	-345486.7	691001.4	0.29	0.464
										9	-345501.5	691029.0	27.92	0.000
										8	-345502.6	691029.3	28.18	0.000
										8	-345525.7	691075.3	74.25	0.000
										9	-345524.9	691075.9	74.77	0.000
										7	-345544.1	691108.2	107.08	0.000

*The shaded boxes indicate the variables included in the model. Models with darker shading represent the confidence set, which has a likelihood of more than 95% to encompass the variables of the best-approximating model (based on AICcWt). K refers to the number of parameters. LL represents the log-likelihood. ΔAICc indicates the difference in corrected AIC compared to the top-ranked model (values < 2 suggest informational equivalence). AICcWt denotes the relative weight of the AICc for a specific model within the entire set of models (values approximate the likelihood that a given model is the best among those considered).

### 3.4 Effect of preoperative radiotherapy on OS in various subgroups

**Figure 3:**
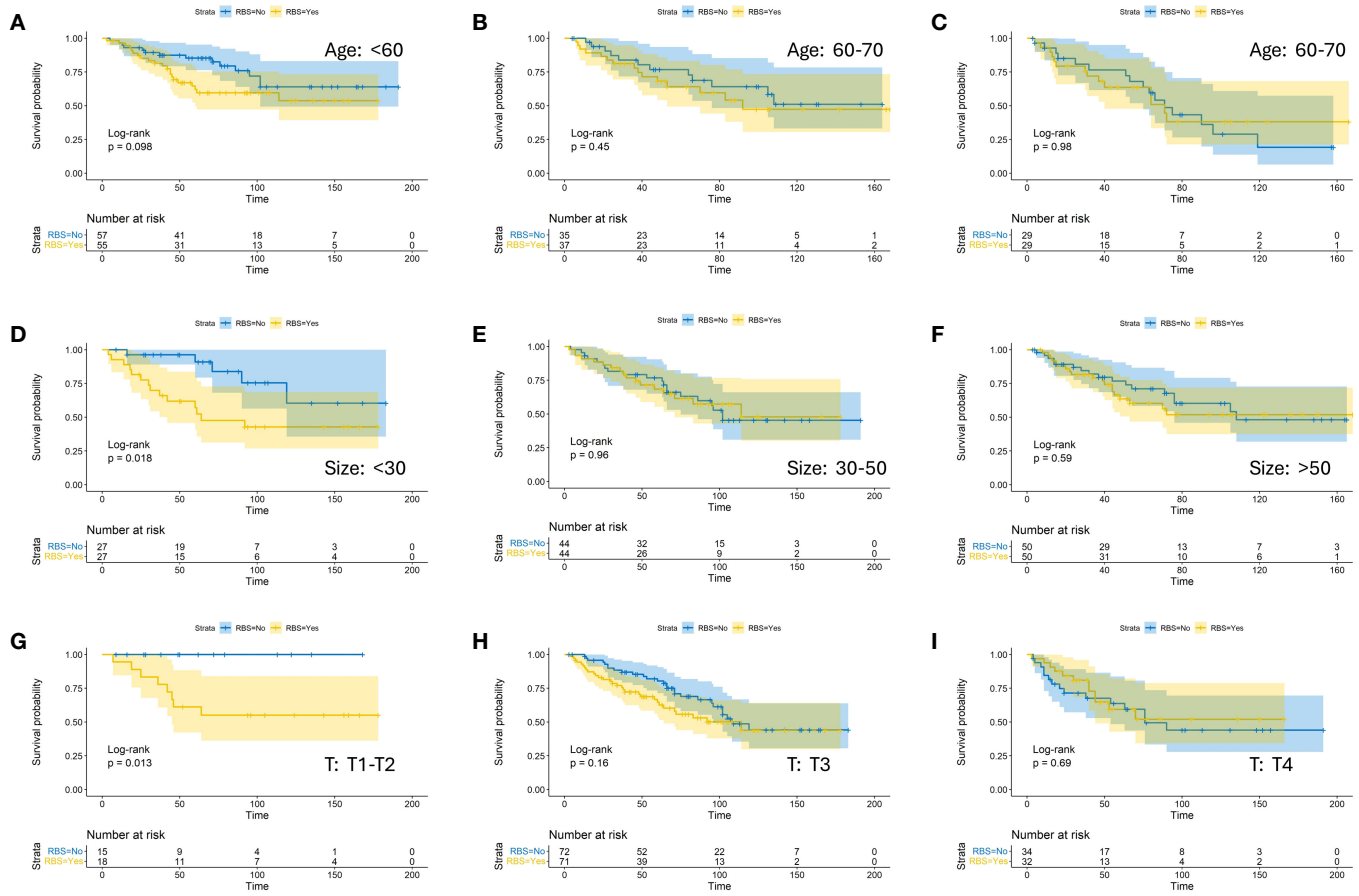
Kaplan-Meier estimates of OS with corresponding 95% confidence intervals for patients in the RBS and none-RBS groups after PSM: Stratified by Age **(A–C)**, Tumor Size **(D–F)**, and T-Stage **(G–I)**.

**Figure 4:**
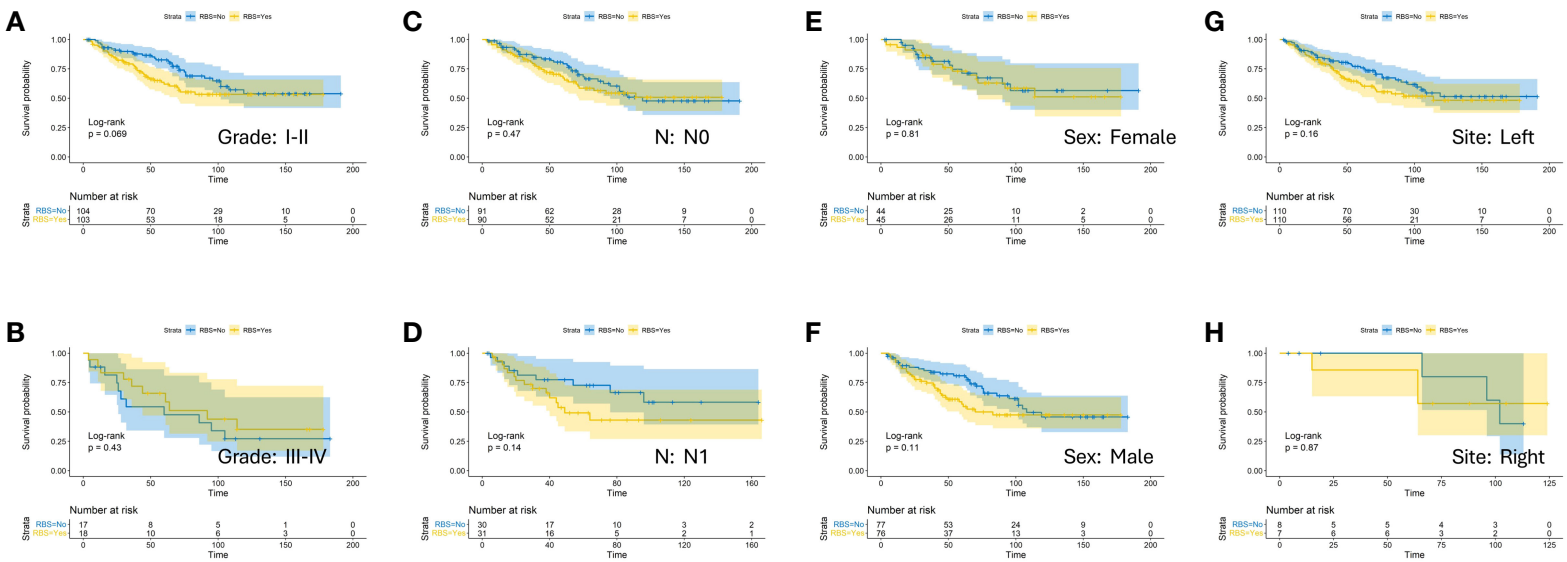
Kaplan-Meier estimates of OS with corresponding 95% confidence intervals for patients in the RBS and none-RBS groups after PSM: Stratified by Grade **(A, B)**, N-Stage **(C, D)**, Sex **(E, F)** and Tumor Site **(G, H)**.

To investigate the impact of RBS on the prognosis of CA patients with specific clinical characteristics more precisely, we stratified patients within each factor’s subgroup in the aforementioned models and compared the OS between RBS and No-RBS groups before and after PSM. The results showed that in the subgroups with tumor diameter <30mm (**Figure 3D**) and T staging of T1-T1 (**Figure 3G**), patients who received RBS had significantly better prognosis than those without RBS (p=0.018; p=0.013). Meanwhile, in the subgroups of age <60 years (**Figure 3A**) and Grade I-II (**Figure 4A**), non-RBS patients exhibited a better prognosis for a significant duration of time, although the differences were not statistically significant (p=0.098; p=0.069). In the remaining subgroup analyses, although there was no statistically significant difference in OS between the two groups, some subgroups still showed a trend of better OS in the RBS group compared to the No-RBS group (**Figures 3**, **4**).

## 4 Discussion

Currently, there is limited research focusing on whether preoperative radiotherapy provides benefits for patients with stage III CA, and there are no clear guidelines to provide guidance in this regard. Consequently, clinicians face challenges in making appropriate decisions during clinical management, and some physicians tend to lean towards the use of preoperative radiotherapy in patients with locally advanced disease (13–17). In this study, we created a maximally balanced cohort of baseline covariates using propensity score matching and investigated the impact of preoperative radiotherapy on survival. Based on the statistical analysis results, we found that although the incidence rates were higher in the elderly population, females, and those with the primary site located in the right colon, clinicians tend to preferentially administer preoperative radiotherapy to younger individuals, males, and those with the primary site in the left colon. This preference may be due to considerations of patient tolerance to radiation and the operability of the site (18). However, our results show that preoperative radiotherapy does not improve the overall survival of patients with stage III CA. Cox models further confirm that preoperative radiotherapy is not a significant prognostic factor for patients with stage III CA. Regardless of differences in T staging, N staging, differentiation grade, age, sex, tumor location, and tumor size, preoperative radiotherapy does not confer a survival benefit. In fact, in certain specific subgroups, the OS of the RBS group was significantly lower than that of the No-RBS group. This discrepancy may be attributed to the small sample size and high rate of missing data, but it raises questions about whether preoperative radiotherapy not only fails to improve the prognosis of patients with stage III CA but also leads to a decrease in their OS. Furthermore, while many case reports suggest that preoperative radiotherapy may be an effective treatment option for locally advanced colorectal cancer (19), it is important to note that radiotherapy can have negative impacts. For example, radiotherapy can increase the proliferation of residual cells, induce vascular remodeling, and alter cell motility, thereby promoting the regrowth of tumor cells (20). Additionally, preoperative radiotherapy increases the risk of developing subsequent secondary primary tumors in patients (21) and is associated with an increased incidence of anastomotic leakage after surgery (22). The Intergroup 0130 study also indicated that patients who received combined chemoradiotherapy were more likely to experience toxic reactions such as leukopenia and nausea compared to those receiving chemotherapy alone (23).

We have observed that previous studies have indicated that preoperative neoadjuvant radiotherapy can contribute to an increased rate of pathological complete response (pCR) and overall survival (OS) in locally advanced colon cancer (24–27). However, these findings seem to differ from the conclusions drawn in our study. We speculate that this discrepancy may be attributed to several factors. Firstly, Huang et al.’s study focused on patients with T4N2M0 colon cancer, and their study endpoint was 5-year OS, which differs from our study in terms of patient population and research objectives. Additionally, Wang et al.’s study considered chemotherapy regimens concurrently, but they did not conduct a controlled study comparing two cohorts, and their sample size was relatively small. It is worth noting that some scholars argue that adjuvant radiotherapy is not commonly used as definitive treatment for colon cancer (28), although their study primarily focused on postoperative adjuvant radiotherapy (29).

In general, this study incorporated the latest data from a multicenter study with a large sample size. Propensity score matching (PSM) was employed to mitigate potential biases caused by confounding factors, and long-term overall survival (OS) served as the study endpoint, providing robust evidence for clinical decision-making in treatment selection. However, like any SEER-based study, there are limitations to consider. Firstly, the SEER database does not include information on patients’ physical fitness or reasons for not receiving adjuvant radiotherapy. Secondly, the SEER database lacks data on preoperative radiotherapy, including clinical target volume and radiation protocols, which weakens the conclusions of the current study. Additionally, whether patients experienced toxic reactions after radiotherapy remains unknown. Thirdly, due to the non-routine inclusion of adjuvant radiotherapy in the preoperative treatment of colon cancer patients, even though we included data from all patients over a 15-year period, the sample size of the study may still be insufficient. Lastly, there may be variations in the acceptance rate of preoperative radiotherapy among different healthcare regions. Therefore, we hope that future randomized multicenter clinical trials on a global scale can provide further validation in this regard.

## 5 Conclusion

Based on our study findings, we conclude that preoperative radiotherapy does not improve the long-term prognosis of patients with stage III CA. In fact, in certain patient populations with specific clinical characteristics, preoperative radiotherapy may even lead to a decrease in OS.

## Data availability statement

Publicly available datasets were analyzed in this study. This data can be found here: https://seer.cancer.gov/.

## Ethics statement

The requirement of ethical approval was waived by Surveillance, Epidemiology, and End Results database for the studies involving humans because Surveillance, Epidemiology, and End Results database. Since the data from SEER are publicly available and deidentified, this study was exempt from local institutional review board review. The studies were conducted in accordance with the local legislation and institutional requirements. The participants provided their written informed consent to participate in this study.

## Author contributions

JX: Conceptualization, Methodology, Software, Visualization, Writing – original draft. XN: Data curation, Investigation, Supervision, Validation, Writing – review and editing.
